# Early Use of the NMDA Receptor Antagonist Ketamine in Refractory and Superrefractory Status Epilepticus

**DOI:** 10.1155/2015/831260

**Published:** 2015-01-12

**Authors:** F. A. Zeiler

**Affiliations:** Section of Neurosurgery, Department of Surgery, University of Manitoba, Winnipeg, MB, Canada R3A 1R9

## Abstract

Refractory status epilepticus (RSE) and superrefractory status epilepticus (SRSE) pose a difficult clinical challenge. Multiple cerebral receptor and transporter changes occur with prolonged status epilepticus leading to pharmacoresistance patterns unfavorable for conventional antiepileptics. In particular, n-methyl-d-aspartate (NMDA) receptor upregulation leads to glutamate mediated excitotoxicity. Targeting these NMDA receptors may provide a novel approach to otherwise refractory seizures. Ketamine has been utilized in RSE. Recent systematic review indicates 56.5% and 63.5% cessation in seizures in adults and pediatrics, respectively. No complications were described. We should consider earlier implementation of ketamine or other NMDA receptor antagonists, for RSE. Prospective study of early implementation of ketamine should shed light on the role of such medications in RSE.

## 1. Introduction

Refractory status epilepticus (RSE) is defined as either generalized or complex partial status epilepticus (SE) that fails to respond to first and second line therapies. Superrefractory status epilepticus (SRSE) is SE that remains unresponsive despite 24 hours of therapy with general anesthesia [1, 2]. Both RSE and SRSE pose significant challenges for the managing intensivist.

The incidence of RSE varies in patients with SE, up to 40% [3]. Overall mortality rates for RSE have been reported to approach upwards of 50% [1, 3, 4]. With the development of RSE, literature suggests a significant impact on length of hospital stay and functional morbidity [4]. Similarly, poor functional outcome in patients with RSE has been reported in upwards of 75% [3]. In comparison, data for outcome in the SRSE population is scarce. However, outcomes seem similar to the RSE population with a recent review of 1168 patients displaying only a 35% recovery to preadmission baseline [5].

There exists a race against time for control of epileptic activity in the RSE/SRSE patient, in order to preserve cortical function and reduce morbidity/mortality. In fact the duration of uncontrolled SE has been demonstrated to correlate with outcome [6]. Thus the pharmacotherapy utilized in the management of SE should be such that adequate seizure suppression and sustained control are obtained, in order to prevent the transition from standard SE to RSE or SRSE.

However despite the best intentions, and not uncommonly, standard frontline antiepileptic drugs (AEDs) fail to control or reduce seizure activity once seizures approach the 30-minute mark [7]. Specific alterations in receptors and molecular transporters at the level of the neuron and blood-brain-barrier (BBB) are attributed to the pharmacoresistance patterns commonly seen in RSE/SRSE.

With these receptor alterations comes the need for novel therapeutic targets in the treatment of RSE/SRSE. Targeting the n-methyl-d-aspartate (NMDA) receptor may be one of these novel and effective means of achieving seizure control and providing neuroprotection in difficult cases of RSE/SRSE.

The following review provides an analysis of common pharmacological targets in RSE/SRSE, receptor changes leading to pharmacoresistance patterns, and the evidence for ketamine/NMDA receptor antagonists for RSE/SRSE, with a focus on the potential target population, dosing, concerns, and the role for early administration.

## 2. Materials and Methods

We performed a review of recent literature surrounding RSE/SRSE, summarized current receptor targets for commonly utilized AEDs, and provided an argument for early consideration of ketamine in RSE/SRSE.

## 3. Results/Discussion

### 3.1. Receptor Pharmacology

#### 3.1.1. Standard AED Targets

Benzodiazepines are arguably the most common frontline AED utilized in SE. With intermittent bolus dosing or via continuous infusions, benzodiazepines typically form the cornerstone of seizure management [8, 9]. The GABA agonist effects lead to cortical inhibition and reduction of epileptogenicity and lateral spread. Recent review indicates class Ia, level A evidence for the use of lorazepam and midazolam in the emergent treatment of seizures [8].

Other GABA mediated medications include valproate, propofol, barbiturates, clonazepam, clobazam, vigabatrin, and topiramate [9], all with varying levels of evidence in the emergent management of seizures and in RSE.

Sodium channel blockers are also commonly utilized as AEDs. Such drugs include phenytoin, carbamazepine, lamotrigine, oxcarbazepine, zonisamide, and rufinamide [8, 9]. Currently in the setting of RSE, sodium channel blockers such as phenytoin only carry class IIb, level C evidence for seizure control.

Calcium channel blockers, such as gabapentin and pregabalin, have been implemented for management of seizures. Their role in RSE and SRSE is currently undefined.

#### 3.1.2. RSE/SRSE Related Receptor Alterations

First, downregulation in the gamma-aminobutyric acid type A (GABA_A_) receptors leads to a depletion of available receptors for commonly utilized benzodiazepines or GABA mediated AEDs. Furthermore, subunit alterations in the GABA_A_ receptor lead to impaired binding of both GABA and GABA mediated AEDs resulting in the GABA resistant state commonly seen in RSE [10, 11].

Second, upregulation of p-glycoprotein molecular transporters at the level of the BBB occurs with status epilepticus approaching 20 to 30 minutes in duration. These transporters export phenytoin and phenobarbital molecules, both commonly utilized frontline medications in RSE [12]. Subsequently, the efficacy of phenytoin and phenobarbital is greatly diminished.

Finally, prolonged status epilepticus leads to upregulation of n-methyl-d-aspartate (NMDA) receptors. Glutamate mediated activation of these receptors occurs, promoting intracellular calcium influx and subsequent excitotoxicity that further potentiates epileptogenicity [10, 13].

#### 3.1.3. RSE/SRSE Nonreceptor Mediated Mechanisms of Pharmacoresistance

Other mechanisms have been postulated for the pharmacoresistance patterns seen in RSE/SRSE. Proinflammatory mediators may play a key role in this resistance via alterations in the BBB permeability and neuronal damage [7, 14, 15]. Both of these inflammatory mediated changes lead to AED resistance and excitotoxicity that can further potentiate SE/RSE/SRSE. Thus, a role for anti-inflammatory agents and neuroprotective agents, such as NMDA antagonists [16, 17], has been suggested.

Finally, the severity of SE is a predictor of resistance. The magnitude and severity of the underlying pathology for SE impact the effectiveness of the AEDs utilized and need to be taken into account [7].

### 3.2. Rationale and Evidence for NMDA Receptor Antagonists in RSE

#### 3.2.1. Rational

The majority of current AEDs function via GABA, sodium channel, or calcium channel mediated mechanisms [8, 9], as previously outlined. Given the aforementioned alterations in cerebral receptor and transporter functions in RSE/SRSE [7], in addition to the nonreceptor mediated mechanisms of pharmacoresistance, the efficacy of the majority of AEDs is impacted, and thus there exists a need for novel therapeutic targets. Targeting the NMDA receptor provides such a novel approach.

The use of NMDA receptor antagonists for SE, such as ketamine, provides a few benefits. First, NMDA receptor antagonists target a receptor known to be upregulated during SE/RSE/SRSE and one that contributes to excitotoxicity [7, 13, 16–18]. Second, NMDA receptor antagonists provide a degree of neuroprotection even after SE [16, 18]. This neuroprotective effect has even been studied within the traumatic brain injury literature [19, 20]. Third, in regard to ketamine, this drug is readily available and cheap, allowing for application in a variety of settings. Fourth, the sympathomimetic properties of ketamine in particular afford it vasopressor sparing effects, which reduce the need for vasoactive compounds to counteract the hypotension commonly seen with other intravenous anesthetics used in SE. Finally, the side effect profile in the neurological population, as documented in the literature to date, is low despite some initial concerns about potential neurotoxicity.

#### 3.2.2. Animal Studies

Numerous small animal models of SE have demonstrated the effectiveness of ketamine seizure control/cessation and neuroprotection [21–25]. A variety of different models of SE have been studied to determine the efficacy of ketamine as an AED and neuroprotective agent.

The antiepileptic effects of ketamine in models of SE are well documented. Both rat and guinea pig pilocarpine/soman models of SE have displayed the antiepileptic properties of ketamine robustly [17, 22, 23].

The neuroprotective effect of ketamine in SE models is impressive. Pilocarpine models display the efficacy of ketamine in the reduction of SE related mortality and overall volume of neuronal damage, in comparison to controls [21]. In one rat pilocarpine model, SE induced neuronal death was prevented with ketamine administration in all regions assessed compared to control groups [24].

There are some concerns within the animal literature with the use of NMDA receptor antagonists as neuroprotective agents. Neuronal vacuolization and subsequent neuronal necrosis have been reported with escalating doses of the NMDA receptor antagonist MK(+)-801 in rat retrosplenial cortex [26]. In addition, other models have displayed induced neuronal apoptosis in rat traumatic brain injury models related dizoclipine and other NMDA receptor antagonists [27].

These concerns have yet to be replicated in human subjects. However the potential for a similar response to high dose or long term application of NMDA receptor antagonists exists.

#### 3.2.3. Human Studies

To date the only NMDA antagonist utilized for treating seizures and RSE is ketamine. The majority of these patients fit criteria for SRSE, given the duration of uncontrolled SE. With regard to the efficacy of ketamine as an antiepileptic, a recent systematic review identified 23 studies utilizing ketamine for RSE in both adult and pediatric populations [28].

Twenty-two of the 23 studies identified were original studies focusing on the use of ketamine as an antiepileptic in the setting of RSE. Three prospective cohort studies were identified, with the remaining articles being retrospective. There was a mean of 7 patients per study. The number of AEDs on board prior to ketamine varied from 1 to 11, with most having trialed these medications for a couple of weeks prior to considering ketamine.

Of the 110 adult patients identified, 56.5% responded to ketamine administration by cessation of their status epilepticus. The duration of treatment prior to ketamine was 16 hours to 140 days. The usual dosing was a bolus of 0.5 to 5 mg/kg, followed by a continuous infusion of 0.12 to 10 mg/kg/hr, with duration of treatment varying from 2 hours to 27 days. The commonly reported trial of ketamine lasted around 7 days, with most responding within 48 to 72 hours of initiation.

Similarly, within the 52 pediatric patients identified, 63.5% had cessation of their RSE. A variety of ketamine dosing regimens were utilized within the studies identified. Bolus dosing was reported up to 3 mg/kg, with infusions reported up to 10 mg/kg/hr for duration of 6 hours to 27 days.

Of interest, no major complications were described. Two patients had nonhemodynamically significant arrhythmias, while one displayed hypersalivation. Patient outcomes were poorly documented and not the focus of the majority of literature identified in the systematic review.

The conclusion of this review indicated Oxford level 4 and Grade D evidence for the use of ketamine in RSE. Despite this low level of evidence for its use in RSE/SRSE, ketamine should definitely be tried in those patients with refractory status epilepticus where other anaesthetics have failed or are causing serious cardiac depression or circulatory compromise.

### 3.3. Argument for Early Administration of NMDA Antagonists

One major question arose from the review and still remains: Would ketamine/NMDA receptor antagonists be more effective if utilized earlier in RSE/SRSE? The simple answer is that we do not know. However, one could extrapolate that with the reasonable success obtained in the cases summarized in the systematic review and with earlier implementation of NMDA receptor antagonism, less glutamate mediate excitotoxicity would occur, and potentially status epilepticus will be less refractory. This has yet to be seen.

Why not utilize ketamine/NMDA receptor antagonists earlier in RSE/SRSE? It is difficult to find one good reason not to. When should we trial these drugs in RSE/SRSE? Is it potentially after the failure of the initial benzodiazepine and phenytoin load? Is it after the failure of the initial intravenous sedative agent? This is still up for debate.

### 3.4. Recommendations in Early Implementation of NMDA Receptor Antagonists for RSE/SRSE

#### 3.4.1. Potential Populations of Interest

There really has not been a population identified where ketamine would be contraindicated for use in RSE/SRSE. The populations that would be of interest for an early trial of ketamine would be those that failed to respond to first and second line agents. These agents should include both a GABA agonist and sodium channel blocker, so as to attack the problem from the three main receptors at play in SE.

A reasonable goal would be to implement ketamine within 24 to 48 hours of SE onset, right after the failure of the first trial with one of the anaesthetics of first choice (midazolam, propofol, and thiopentone/pentobarbital). It is quite possible that the inflammatory mediated BBB and neuronal changes that occur with ongoing seizures will have an impact on the effect of ketamine. Thus starting it 1 or 2 weeks into the treatment of SE may be too late and account for the response rates described in the systematic review [28].

#### 3.4.2. Dosing Regimens

Based on the studies identified in the systematic review, ketamine is the NMDA receptor antagonist medication to implement currently. The dose should include a bolus dose around 3 mg/kg, since this is the middle of the range described in the literature. Continuous infusion should follow ranging up to 10 mg/kg/hr, as this is the upper limit described. Finally, duration of treatment should be up to 7 days. Most patients studied responded within 48 to 72 hours of ketamine initiation.

What about other NDMA receptor antagonists? Simply we do not have any other studies utilizing them for RSE/SRSE, so we cannot recommend their use at this time.

#### 3.4.3. The Concerns and Populations at Risk

Complications related to ketamine administration are of concern. Mild hypertension, arrhythmias, hypersalivation, and hallucinations have all been described with ketamine use. Such complications were few and minor in the review of ketamine for RSE. Despite this, one could suggest avoiding ketamine for RSE/RSE in those with a history of significant cardiac arrhythmias.

One major looming concern, propagated throughout anesthesia and critical care literature, is that of intracranial pressure (ICP) elevation with ketamine administration in the neurologically ill. Though this seemed to be the case in the literature of the 60s and 70s, recent reviews have indicated Oxford level 2b, Grade C evidence against ICP elevations in both adult traumatic [29] and nontraumatic neurological illnesses [30]. Thus, ICP concerns should not be factored into whether or not to start ketamine for RSE/SRSE.

Finally, the impact on patient outcome has yet to be determined and is also concerning. The systematic review failed to shed light on the impact of ketamine on mortality and functional outcomes in the patients treated. To date no formal comments on the impact of ketamine on patient outcome in RSE/SRSE can be made.

### 3.5. Opinion and Future Recommendations

A lot of questions still exist surrounding the utility of NMDA receptor antagonists in SE/RSE/SRSE. The one question we can possibly answer is, Should we consider ketamine earlier in RSE/SRSE? The answer is yes. This is not just based on its potential benefits for NMDA mediated epileptogenicity in RSE/SRSE, but the answer “yes” also stems from the vasopressor sparing anesthetic effects, its analgesic effects, and lack of significant adverse effects documented in this patient population.

We should prospectively study early use of ketamine/NMDA receptor antagonists for RSE/SRSE. Multicenter prospective study of early implementation of NMDA receptor antagonists needs to occur in order for us to understand the role of these compounds in the management of RSE/SRSE. As further prospective studies utilizing NMDA receptor antagonists in refractory seizures emerge, further light will be shed on this potential weapon in our arsenal in the treatment of RSE/SRSE.

## 4. Conclusions

Given receptor changes during RSE/SRSE we need to explore other targets for AED therapy. Ketamine is an AED that provides a novel target with minimal documented side effects. Future studies utilizing ketamine early in RSE/SRSE need to be conducted.
